# Rapid serum tube technology overcomes problems associated with use of anticoagulants

**DOI:** 10.11613/BM.2019.030706

**Published:** 2019-10-15

**Authors:** Kong-Nan Zhao, Goce Dimeski, John de Jersey, Lambro A Johnson, Michael Grant, Paul P Masci, Martin F Lavin

**Affiliations:** 1 Faculty of Medicine, University of Queensland, Translational Research Institute, Brisbane, Australia; 2 Chemical Pathology, Princess Alexandra Hospital, Brisbane, Australia; 3 School of Chemistry and Molecular Biosciences, University of Queensland, Brisbane, Australia; 4 Q-Sera Pty Ltd, Level 9,31 Queen St, Melbourne, Australia

**Keywords:** prothrombin activators, serum, anticoagulants, analytes

## Abstract

**Introduction:**

Failure to obtain complete blood clotting in serum is a common laboratory problem. Our aim was to determine whether snake proth-rombin activators are effective in clotting blood and producing quality serum for analyte measurement in anticoagulated patients.

**Materials and methods:**

Whole blood clotting was studied in a total of 64 blood samples (41 controls, 20 Warfarin patients, 3 anticoagulated patients using snake venom prothrombin activator (OsPA)) with plain tubes. Coagulation was analysed using a visual assay, Hyland-Clotek and thromboelastography. Healthy control blood was spiked with a range of anticoagulants to determine the effectiveness of OsPa-induced clotting. A paired analysis of a Dabigatran patient and a control investigated the effectiveness of the OsPA clotting tubes. Biochemical analytes (N = 31) were determined for 7 samples on chemistry and immunoassay analysers and compared with commercial tubes.

**Results:**

Snake venom prothrombin activators efficiently coagulated blood and plasma spiked with heparin and commonly used anticoagulants. Clotting was observed in the presence of anticoagulants whereas no clotting was observed in BDRST tubes containing 3 U/mL of heparin. Snake venom prothrombin activator enhanced heparinised blood clotting by shortening substantially the clotting time and improving significantly the strength of the clot. Comparison of 31 analytes from the blood of five healthy and two anticoagulated participants gave very good agreement between the analyte concentrations determined.

**Conclusions:**

Our results showed that the snake venom prothrombin activators OsPA and PtPA efficiently coagulated recalcified and fresh bloods with or without added anticoagulants. These procoagulants produced high quality serum for accurate analyte measurement.

## Introduction

The presence of prothrombin activators (PA) in the venoms of snakes, including *O. scutellatus* (Australian coastal taipan, OsPA) and *P. textilis* (eastern brown snake, PtPA) causes rapid coagulation of blood (*1*, *2*). These prothrombin activators do not require clotting co-factors such as calcium and phospholipids which are required by the mammalian prothrombinase complex (*3*, *4*). In a recent report we demonstrated that OsPA, had strong coagulation activity in clotting fresh whole blood and also recalcified citrated whole blood from normal patients (*5*). We demonstrated that the mechanism of action involved rapid and sustained generation of large amounts of thrombin, followed by the efficient conversion of fibrinogen to fibrin for complete and stable clotting. In that study we also showed that the quality of the serum produced was appropriate for standard analyte determinations carried out in pathology laboratories. Comparison between OsPA-containing tubes and commercial tubes showed that the values for analyte concentrations were in excellent agreement for up to seven days storage. While efficient clotting and high quality analyte determination was achieved under these conditions with normal individuals, the efficacy of OsPA in clotting blood in the presence of anticoagulants or from patients on anti-coagulation therapy presents a further challenge for preparation of quality serum in a diagnostic setting.

The anticoagulant most commonly used in cardiac, renal dialysis and liver disease patients is heparin which functions by forming a complex with antithrombin that normally binds tightly to thrombin to inhibit blood clotting and thus interfere with the ability to prepare serum samples and consequently with the accuracy of blood diagnosis (*6*-*8*). As an approach to addressing this problem, thrombin-containing rapid serum tubes were first introduced in the US and EU markets by Becton Dickinson (BDRST) to speed up the clotting process in order to hasten urgent analyte testing (thrombin based tubes having being previously launched in Japan). The correlation between analyte determination in serum prepared in these tubes and plain tubes was generally good although there were some differences (*9*). A more recent evaluation showed that results for renal, liver, cardiac, thyroid, and prostate biochemical markers were comparable between RSTs and SSTs (BD advanced tube containing silica clot activator). However, analyte stability on serum storage at 4 °C showed some deterioration for bicarbonate, electrolytes and albumin over a period of four days in thrombin-containing tubes (*10*). While the RST tube provides a suitable alternative to lithium heparin plasma tubes for most patients, latent clotting continued to occur in samples collected from patients on high concentrations of anticoagulants (*8*). The manufacturer does not recommend BDRST tubes for heparinised blood. More recently Greiner Bio-One (GBO) has produced a fast clotting tube (BCA Fast Clot; GBOFC) which also contains thrombin as well as a clot activator (*11*). Overall, equivalent results to those obtained with the BDRST tube were observed for the GBOFC tubes (*12*). Thus, the aim here is to determine whether snake venom prothrombin activators (OsPA and PtPA) are effective in clotting blood and producing high quality serum for accurate analyte measurement in the presence of anticoagulants.

## Materials and methods

### Subjects and study design

**Figure 1:**
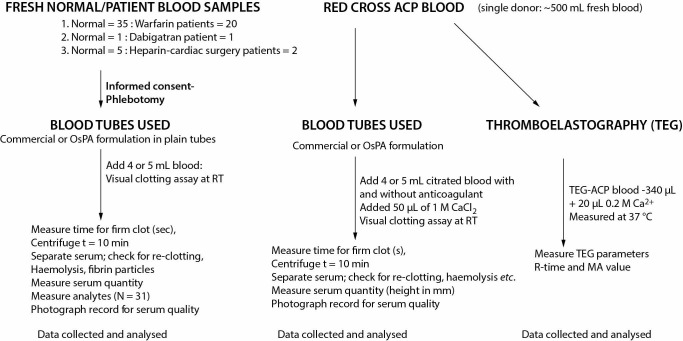
Flow chart of normal and anticoagulated blood processing to assess quality serum. RT – room temperature. R -reaction time. MA – maximal amplitude.

This study was designed to determine the effectiveness of OsPA and PtPA in clotting human blood with or without anticoagulants to produce high quality serum for accurate analyte measurement. The snake venoms were collected under a National Parks and Wildlife permit W4/0026/01/SAA and supplied by Venom Supplies (Tanunda, SA, Australia). The OsPA was purified from snake *O. scutellatus* venom while PtPA was purified from snake *P. textilis* venom (*1*, *13*). Both prothrombin activators were stored as described previously (*1*, *13*). *O. scutellatus* venom prothrombin activator concentrations at 0.03125 to 2 µg/tube were used for plasma clotting assays and at 1-10 µg/tube for blood clotting assays. A total of 500 blood collection tubes with different concentrations of OsPA were prepared for investigating the effectiveness of OsPA in clotting recalcified citrated and fresh human blood. Citrated whole human blood either from the Australian Red Cross Blood Service (ARCBS), Brisbane or the Princess Alexandra Hospital (PAH) Blood Bank was added to OsPA-containing tubes and clotting time was observed visually in samples containing recalcified citrated whole blood and blood samples spiked with different anticoagulants. In order to determine the physical properties of the clot, thromboelastography (TEG) was performed for blood sample clotting in both OsPA-containing tubes and BDRST tubes. In addition, to investigate this in a clinical scenario, fresh blood samples from 35 healthy participants and 20 patients on warfarin treatment (international normalised ratio range (INR) 1.0-3.6) were recruited. Two healthy participants with INR 1.0-1.1 and two warfarin patients with INR of 2.5 and 4.7 were also recruited for plasma clotting assays by visual observation and TEG. In addition, 1 patient on Dabigatran therapy (110 mg/*per* tablet twice a day), 6h post-treatment and one healthy participant were recruited for fresh whole blood clotting assays (Supplementary table 1). Biochemical analytes in serum produced in OsPA- and PtPA-containing tubes were determined in seven participants (5 healthy and 2 anticoagulated participants) on chemistry immunoassay analysers and compared with two commercial GBO tubes (Rapid serum (Serum/GBO) and Plasma/Heparin tubes). The flowchart of the study design is shown in Figure 1.

Human research ethics approval for this study using purified snake venom fractions was obtained from Metro South Human Research Ethics Committee and The University of Queensland Human Ethics Committee: HREC Reference number: HREC/08/QPAH/005. The project was carried out in compliance with the World Medical Association Declaration of Helsinki regarding the ethical conduct of research on human subjects. All subjects participated in study signed informed consent.

### Tube preparations for visual recalcified citrated whole blood clotting and biochemical analytes assay

Greiner Bio One (Kremsmunster, Austria) ‘no additive’ white top plain blood collection tubes without separator were coated with 20 µL of hydrophilic surfactant (2.41 g/L) (Dow Corning, Freeland, MI, USA) before use and employed throughout the study and called GBO White Top tubes. For the GBO White Top tubes designated “wet”, an aliquot of OsPA working solution in Hepes buffer was added prior to the blood sample for clotting assays as quality controls of OsPA activity in all experiments. For the GBO White Top tubes designated “dried”, aliquots for OsPA solution in tubes were dried at 25-30°C using a Genevac (SP Scientific, UK) vacuum drier for 30 min to make dried OsPA-containing tubes that mimic commercial blood collection tubes used in experiments. A total of 500 dried OsPA–containing tubes were prepared, which were divided into five groups based on the OsPA concentration at 1, 2, 3, 5 and 10 µg OsPA/tube prepared for different experiments. Thirty PtPA–containing tubes at 1 µg PtPA/tube were also prepared for the biochemical analyte measurement experiments. The dried OsPA- and Pt-PA-containing tubes were sealed with laboratory wrap and kept at room temperature for 30-90 days under low vacuum with a drying agent in a desiccator. Becton Dickinson (BD) red top no additive plain blood collection tubes (BD, Franklin Lakes, USA), BD rapid serum tubes (BD, Franklin Lakes, USA), were used as controls for both visual clotting assays and TEG. In addition, both GBO lithium heparin plasma tube (GBO plasma/heparin, Kremsmunster, Austria) and GBO serum tube (serum/GBO, Kremsmunster, Austria) were used for biochemical analyte determination experiments without addition of prothrombin activator.

### Comparison of blood clotting activity between OsPA and BDRST

Visual blood clotting assays were also carried out to compare the activity of OsPA-containing tubes with that of BDRST tubes in clotting whole blood samples with or without four anticoagulants including heparin, warfarin, Rivaroxaban and Dabigatran at different doses in duplicate. In parallel, TEG assays were carried out to compare the activity of OsPA-containing tubes with BDRST tubes in clotting whole blood samples containing heparin and warfarin.

### Chemical agents

Anticoagulants including DBL^TM^ heparin sodium (5000 IU/mL, Hosprira, Melbourne, Australia), Fondaparinux (heparin analog Arixtra^TM^, Sanofisynthelabo, Australia), Low Molecular Weight Heparin (Clexane^TM^, Aventis, Australia), Rivaroxaban (Xarelto^TM^, Bayer, Australia) and Dabigatran etexilate Pradaxa, Boerhinger Ingelheim, Germany) were used in experiments. DBL^TM^ heparin sodium was at 5000 IU/mL solution for directly preparing heparinized blood. Fondaparinux (Arixtra^TM^) at 1.5 mg/0.3 mL solution for injection in a pre-filled syringe and Clexane at 80 mg/0.8 mL solution (anti-Xa: 8000 IU) were directly used for spiking blood. Rivaroxaban (Xarelto^TM^ 20 mg/tablet) was extracted from the tablets by dissolving in dimethyl sulfoxide (DMSO) at a stock concentration of 10 mg/mL. The thrombin inhibitor, Dabigatran etexilate (Pradaxa^TM^) 110mg/tablet used in experiments was dissolved in high performance liquid chromatography (HPLC) grade methanol to prepare a stock solution at 1 mg/mL according to a previously published method to prepare the active metabolite from the pro-drug dabigatran using commercial tablets (*14*, *15*). Both Rivaroxaban and Dabigatran etexilate stock solutions were used for spiking blood at different concentrations based on the requirements in the experiments.

Other chemical reagentsused for the study were S-2238 substrate from Werfen (Artarmon, Australia) and purified human prothrombin from United Bioresearch (Dural, Australia). DBL^TM^ heparin sodium was from Hospira (Melbourne, Australia). Human thrombin and p-nitro aniline were from Sigma (Aldrich, USA).

### Blood sampling and anticoagulants-spiked blood sample preparation

Citrated whole human blood from either ARCBS or PAH was used for spiking with five anticoagulants including heparin, Rivaroxaban, Arixtra, Clexane and Dabigatran for determining OsPA clotting activity, respectively. The citrated whole blood was aliquoted into a set of 50 mL polypropylene conical tubes (Falcon A corning Brand, Tamawipas, Mexico) with each tube containing 30 mL of blood spiked with DBL^TM^ heparin sodium at 0, 1, 2, 3, 4, 5, 10 and 20 U/*per* mL blood dependent on the experimental requirements. The citrated whole blood was also aliquoted into four sets of 50 mL polypropylene conical tubes labeled as Sets A, B, C and D, with individual tubes containing 30 mL of blood. Set A contained seven tubes spiked with Rivaroxaban at 0, 5, 10, 25, 50, 100 and 200 µg/mL blood. Set B contained 10 tubes spiked with Arixtra^TM^ 0, 0.1, 0.2, 0.5, 0.75, 1, 2, 3, 4 and 5 µg/mL of blood. Set C contained six tubes spiked with Dabigatrin etexilate metabolite at 0, 50, 100, 200, 400 and 600 ng/mL blood and set D contained eleven tubes spiked with Clexane at 0, 0.25, 0.5, 1, 2, 4, 6, 8, 10, 15 and 20 U/mL blood.

Blood samples from controls and patients were processed in the same way (Figure 1). Fresh whole human blood was collected from volunteers including healthy participants, patients on Warfarin and Dabigatran treatments, with each volunteer providing 8 mL of fresh blood, which was aliquoted into two tubes, one OsPA containing tube (1 µg OsPA/tube) and the second tube as a control for visually testing OsPA clotting effectiveness. The bloods from healthy participants added into OsPA-containing tubes were labelled as N+OsPA and non-OsPA tube as N-OsPA, while those from warfarin patients were added into OsPA-containing tubes as W+OsPA.

### Citrated plasma preparation, heparin spiking and plasma clotting assay

Citrated blood (3.2% trisodium citrate) obtained from ARCBS was centrifuged at 1500xg for 10 minutes in a refrigerated centrifuge. After centrifugation, citrated plasma was collected into a plastic screw-cap vial. The citrated plasma was spiked with DBL^TM^ Heparin sodium at 0, 1, 2 and 4 U/per mL plasma and used for determining the clotting activity of OsPA. The clotting assay for the citrated plasma spiked with or without heparin in the presence of OsPA was performed using a Hyland-Clotek instrument that measures clotting using a magnetically suspended sphere as described in our recently published paper (*5*). The control clotting time for the recalcified citrated plasma was approxiemately 400 seconds (s).

INR values from patients on Warfarin treatment participating in the study were determined prior to sampling at PAH Pathology using an ACL TOP 500 instrument (Werfen, Artarmon, Australia). International normalised ratio R represents the ratio of PT for a warfarinised patient compared to a normal range of prothrombin time (PT) values. In addition, both PT and activated partial thromboplastin time (aPTT) of a Dabigatran patient and a healthy plasma were determined according to a previously published method (*16*) (Supplementary figure 1).

### Visual recalcified citrated whole blood clotting assay

Clotting of whole blood samples with or without anticoagulants such as Heparin, Rivaroxaban, Arixtra, Clexane, Warfarin and Dabigatran was visually determined in blood collection tubes including OsPA-containing tubes. BDRST, GBO plasma/heparin, serum/GBO and GBO Vacuette® red top blood collection tubes (Kremsmunster, Austria) were used as controls for visual blood clotting assays based on experimental requirements. To each tube, 50 µL of 1 M CaCl_2_ was added, followed by 3.95 mL of citrated normal whole blood; the timer was initiated on the addition of the blood which took 10-15 s. Tubes were recapped immediately after the timer was started and gently tilted every 15 s. Clotting times were estimated visually and recorded when clotting was first observed and when a rigid clot formed. All the blood clotting assays were carried out in duplicate in individual experiments. The clotting times of the recalcified citrated whole blood in both GBO and BD plain tubes in the absence of OsPA were recorded as controls in individual experiments. After the blood clotting in OsPA containing tubes was complete, or at 30-70 min for the tubes containing the blood spiked with high concentrations of different anticoagulants, they were centrifuged at 1500xg for 10 min. All the tubes were kept at room temperature for 18 h and then inverted to check for reclotting and then photographed.

### Thromboelastographic analysis

Thromboelastography was carried out in parallel to determine the physical properties of the blood clot generated in the OsPA-containing tube, compared with the BDRST tube in individual blood samples with or without heparin. The TEG assay was performed on TEG® Haemostasis Analyser 5000 series (Haemscope Corporation, USA) according to the manufacturer’s instructions. Both clotting reaction time (s) and clot strength (MA value, mm) for each blood sample were recorded during blood coagulation. This technology is the only way to determine clotting parameters for re-calcified whole blood.

### Biochemical analyte determination

Fresh whole blood samples collected respectively from seven participants (5 healthy and 2 anticoagulated participants) in syringes were aliquoted into four types of tubes: 1) GBO Plasma/Heparin tube, 2) Serum/GBO tube, 3) GBO plain tube containing OsPA (Serum/OsPA) and 4) GBO plain tube containing PtPA (Serum/PtPA). Serum/OsPA and Serum/PtPA tubes did not contain any additives or gel barrier. While the bloods from all the participants were not clotted at all in Plasma/Heparin tube, they formed rigid clots in Serum/GBO, Serum/OsPA and Serum/PtPA tubes within five minutes to produce high quality serum and obtain faster turn-around-times (TAT). Thus, both plasma and sera prepared from all the four tube types by centrifugation at 1500xg for 10 min were used to determine the concentrations of 31 standard biochemical analytes in a diagnostic setting (Table 1).To minimise cellular impact on serum/plasma analytes, 200 µL of serum or plasma samples were immediately transferred into separate tubes and stored at – 20°C before analyte determination. All analyte determinations were performed on the Beckman DxC800 general chemistry and DxI800 immunoassay analysers (Beckman Coulter, Brea, USA) (*6*).

**Table 1:** Analyte data measured at T_0_ in plasma and serum generated from four types of blood collection tubes (N = 7)

						**Mean differences between pairs (%)**				
**Analyte, unit**	**Serum GBO**	**Serum OsPA**	**Serum PtPA**	**Plasma/Heparin**	**P**	**GBO/OsPA**	**GBO/PtPA**	**GBO/Heparin**	**LCS (%)**	**CAL (%)**
Sodium, mmol/L	138 (138-140)	137 (137-140)	137 (137-139)	139 (138-140)	0.091	1.0	0.6	0	2.8	3.0
Potassium, mmol/L	4.3 (4.1-4.6)	4.0 (4.0-4.4)	4.1 (4.1-4.4)	4.0 (3.9-4.2)	0.395	0.1	0.1	4.9	3.3	5.0
Chloride, mmol/L	103 (102-104)	102 (102-104)	102 (101-103)	103 (102-104)	0.348	1.1	1.0	0.0	3.3	5.0
Carbon dioxide, mmol/L	25.8 (25.6-30)	25.4 (25.2-26.5)	25.0 (24.4-25.6)	26.7 (25.5-28.0)	0.053	4.1	5.1	0.4	4.8	10.0
Glucose, mmol/L	5.2 (5.2-6.0)	5.2 (5.2-6.2)	5.3 (5.2-6.2)	5.3 (5.3-6.3)	0.348	1.1	0.6	- 1.5	4.4	10.0
Urea mmol/L	4.8 (4.3-6.1)	4.7 (4.3-5.9)	4.9 (4.3-6.0)	4.7 (4.4-6.1)	0.710	2.7	0.5	0.7	5.0	10.0
Creatinine, µmol/L	84 (75-94)	84 (73-93)	82 (71-94)	82 (73-91)	0.868	0.5	1.0	1.7	5.5	10.0
Urate, µmol/L	350 (290-430)	340 (290-430)	350 (290-440)	350 (29-440)	0.869	1.8	0.4	0.1	3.8	10.0
Total protein, g/L	68 (67-75)	70 (68-77)	69 (68-77)	71 (71-79)	0.061	1.8	- 0.3	- 4.6	3.5	5.0
Albumin, g/L	41 (40-44)	42 (40-45)	42 (40-45)	41 (40-44)	0.682	- 0.1	- 0.3	0	3.2	5.0
Total bilirubin, µmol/L	15.7 (14-22)	15 (13-23)	15 (13-24)	14 (12-23)	0.948	1.6	- 0.9	5.2	7.0	10.0
Alkaline phosphatase, U/L	64 (62-103)	67 (63-105)	60 (59-108)	61 (60-104)	0.602	- 1.1	0.4	0.4	4.8	10.0
Gamma glutamyltransferase, U/L	24 (14-29)	24 (15-27)	24 (15-29)	25 (19-28)	0.301	- 2.7	- 2.8	- 14.7	7.3	10.0
Alanine transaminase, U/L	35 (21-49)	35 (24-51)	36 (24-50)	34 (23-49)	0.137	- 4.2	1. 3	- 4.4	6.8	10.0
Aspartate aminotranferase, U/L	21 (19-28)	21 (21-25)	21 (21-23)	19 (19-21)	0.173	- 1.9	1.9	9.4	6.2	10.0
Lactate dehydrogenase, U/L	194 (166-276)	195 (164-206)	193 (172-219)	206 (164-218)	0.710	0.3	- 3.1	- 2.2	3.7	15.0
						**Mean differences between pairs (%)**				
**Analyte, unit**	**Serum GBO**	**Serum OsPA**	**Serum PtPA**	**Plasma/Heparin**	**P**	**GBO/OsPA**	**GBO/PtPA**	**GBO/Heparin**	**LCS (%)**	**CAL (%)**
Creatinine kinase, U/L	72 (64-144)	72 (67-144)	69 (67-145)	83 (66-139)	0.266	- 0.3	- 1.2	- 2.1	3.7	15.0
Calcium, mmol/L	2.39 (2.30-2.47)	2.35 (2.29-2.47)	2.38 (2.29-2.47)	2.37 (2.28-2.41)	0.093	1.4	0.7	1.2	3.5	5.0
Phosphate, mmol/L	1.20 (1.19-1.32)	1.21 (1.18-1.31)	1.22 (1.17-1.32)	1.13 (1.10-1.27)	0.088	1.3	- 0.6	4.1	3.8	10.0
Magnesium, mmol/L	0.93 (0.92-0.99)	0.92 (0.89-0.97)	0.95 (0.90-0.97)	0.91 (0.90-0.97)	0.602	2.6	2.1	1.5	4.4	10.0
Cholesterol, mmol/L	5.1 (4.7-6.3)	5.0 (4.6-6.5)	5.0 (4.9-6.5)	5.0 (4.5-6.5)	0.564	0.1	- 1.2	0.3	3.6	10.0
Triglyceride, mmol/L	1.3 (1.1-3.6)	1.9 (1.5-4.2)	2.0 (1.6-4.2)	1.4 (1.1-3.7)	0.046	- 159	- 69.4	- 1.5	5.3	10.0
High density cholesterol, mmol/L	1.19 (1.16-1.24)	1.18 (1.17-1.24)	1.15 (1.14-1.20)	1.24 (1.20-1.26)	0.064	2.6	4.8	- 1.7	5.2	10.0
Iron, µmol/L	12 (10-23)	13 (11-23)	13 (11-23)	12 (10-22)	0.401	- 1.8	- 1.5	0.6	3.7	10.0
Transferrin, g/L	2.44 (2.22-2.95)	2.50 (2.29-2.96)	2.48 (2.28-3.03)	2.37 (2.27-2.99)	0.134	- 0.8	- 1.3	- 0.2	4.8	10.0
C-reactive protein, µg/L	5.0 (5.0-5.0)	5.1 (5.1-5.1)	5.0 (4.9-5.6)	5.0 (5.0-5.0)	0.923	0	4.8	7.1	8.8	10.0
Cortisol, nmol/L	241 (193-354)	224 (164-338)	220 (177-349)	232 (188-334)	0.564	3.4	- 0.7	6.3	6.4	15.0
Free thyroxine, pmol/L	11.8 (10.5-13.6)	12.5 (11.1-15.1)	11.6 (11.3-14.1)	12.1 (11.3-14.3)	0.767	- 1.7	- 0.6	- 0.7	6.5	30.0
Thyroid stimulating hormone, mU/L	1.58 (1.49-1.80)	1.60 (1.46-1.85)	1.60 (1.48-1.73)	1.62 (1.49-1.78)	0.362	4.4	6.0	0.2	7.3	15.0
Ferritin, µg/L	52 (47-236)	51 (49-235)	53 (49-229)	54 (53-240)	0.286	0.9	- 0.3	- 3.8	7.2	15.0
Troponin I, µg/L	0.005 (0.002-0.008)	0.005 (0.002-0.008)	0.01 (0.01-0.019)	0.01 (0.01-0.025)	0.016	- 246	- 343	- 186	8.8	10.0
Serum GBO - serum generated from GBO serum tube. Serum/OsPA - serum generated from GBO plain tube containing OsPA. Serum/PtPA - serum generated from GBO plain tube containing PtPA. Plasma/Heparin - plasma prepared from GBO lithium heparin (GRLH) plasma tube. LSC (%) - least significant change. CAL (%) - critical allowable limit. Trig in Serum/OsPA and Serum/PtPA was significantly higher because OsPA/PtPA were stored in Hepes buffer containing 50% of Glycerol. Analysis was performed on the Beckman DxC800 general chemistry and DxI800 immunoassay analysers (Beckman Coulter, Brea, CA, USA) in Chemical Pathology plasma prepared from GBO lithium heparin (GRLH) plasma tube (Plasma/Heparin), Pathology Queensland, Princess Alexandra Hospital. GBO - Greiner Bio-One. OsPA - prothrombin activator complex from the venom of *O. scutellatus*. PtPA - prothrombin activator complex from the venom of *P. textilis*. P shows if there is any significant difference of the analytes among the four serum/plasma samples. P < 0.05 was considered statistically significant.										

### Statistical analysis

Blood and plasma clotting data representing patient and control samples together with numerical values including mean and standard deviation are included in Figure legends. One way ANOVA unequal variance analysis from Excel-2013 software (Microsoft Corporation, Redmond, Washington, USA) was used for statistical analysis of the clotting times for blood between normal-OsPA blood and warfarin-OsPA blood based on a previously published study (*16*). The Fisher exact test was used to determine whether there were non-random associations between two categorical variables. Therefore, this test was used for statistical comparison of both R times and MA values between BDRST and OsPA tubes.

Data of biochemical analytes from seven participants are presented in median and interquartile ranges (Table 1). Non-parametric statistical analysis was used for testing whether there was a significant difference of biochemical analyte measurements among the four serum/plasma samples. Also mean values between two sets of paired data were used for determining whether there was a significant difference of biochemical analyte measurements between sera generated in two blood collection tubes, or between serum produced in serum tubes and plasma produced in heparin tubes using Excel-2013 (Formula-Statistical program). To calculate the acceptable change limit for each assay or the LSC which is considered to be the smallest difference between successive measurements that are real the following equation was used: LSC = 2.77 √CV_biological difference_ + CV _analytical difference_) to determine if analytical differences existed between the paired differences. With the samples being collected at the same time in the different tubes, no biological variation needed to be included in the calculation of the least significant change, therefore LSC = 2.77 √CV_analytical difference_ was employed. The critical allowable limits (CAL) were determined based on a previous publication (*17*).

## Results

### Capacity of OsPA to clot blood in vitro in the presence of anticoagulants used in hospital

**Figure 2:**
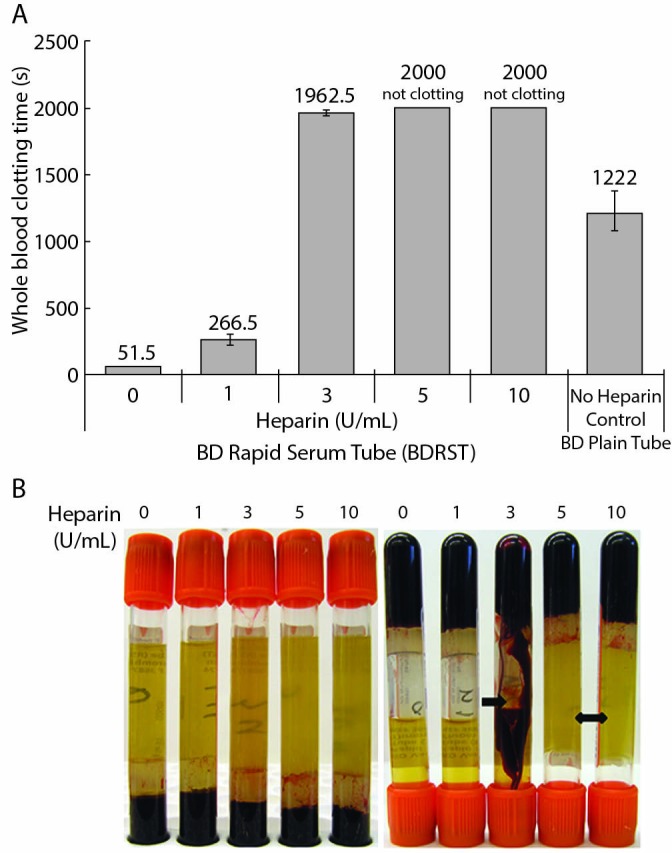
Activity of BDRST tubes in clotting recalcified citrated whole blood spiked with different concentrations of heparin. A) The data are the mean ± SD of the clotting times of duplicate assays. No clots for the bloods containing 5 or 10 U of heparin *per* mL were observed at 2000 s in the BDRST or BD plain tubes. Control (BD plain tube) shows the clotting time (s) for recalcified citrated whole blood without heparin. B) Left panel: Image of blood containing different doses of heparin in thrombin-containing tubes (RST) after the bloods stood in tubes for 2000 s whether they were clotted or not were centrifuged at 1500xg for 10 min. Right panel: Upside-down image of blood containing different doses of heparin in thrombin-containing tubes at 18 h on bench after centrifugation. Two-way arrow - indicates that weak latent clot occurred in tube with blood containing 3U of heparin *per* mL. Arrow - indicates that strong latent clots occurred in tubes with blood containing 5 or 10 U of heparin *per* mL. Image of recalcified citrated whole blood clotted in BD Plain tube is not shown. BD – Becton Dickenson. BDRST- Beckton Dickinson rapid serum tube. SD – standard deviation.

**Figure 3:**
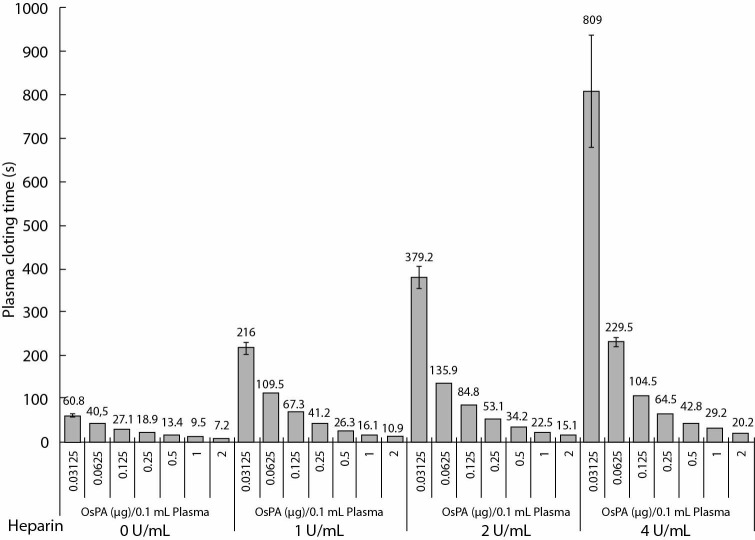
Activity of OsPA in clotting recalcified citrated plasma spiked with different concentrations of heparin. The data are the mean ± SD of duplicate clotting times (N = 2). In the absence of OsPA, the clotting time for the recalcified citrated plasma without heparin was approxiemately 400 s, that has been described in Material and methods section. OsPA - prothrombin activator complex from the venom of *O. scutellatus*. SD – standard deviation.

**Figure 4:**
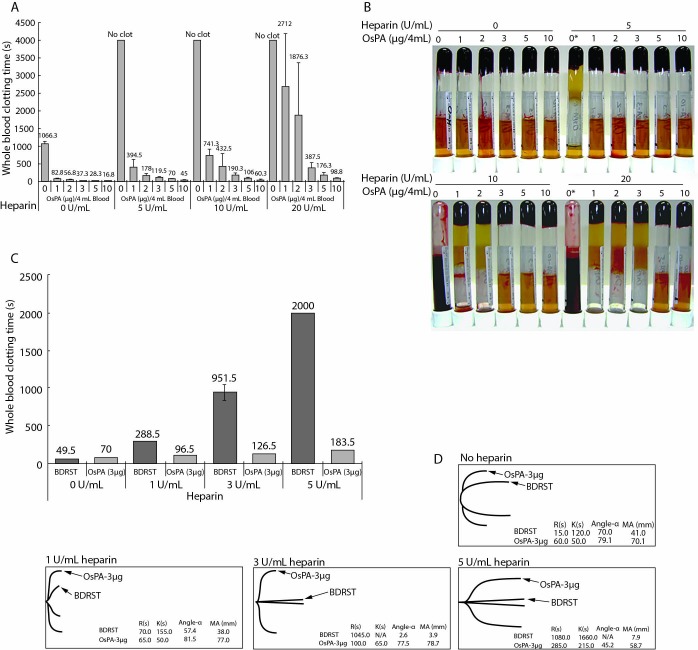
Capacity of OsPA to clot heparinised blood. A) Clotting activity of OsPA in recalcified citrated whole blood spiked with different concentrations of heparin. The data are the mean ± SD of eight clotting times (N = 8) from four experiments. B) Clotting patterns of the heparin-spiked blood with or without OsPA. The blood collection tubes with blood clots at 4000 s were centrifuged at 1500xg for 10 min and then kept on the bench for 18 h. Images were taken of the inverted clot-containing tubes. *indicated that no clots were formed in the heparinised bloods without addition of OsPA at 4000 s before centrifugation. No re-clotting was observed in blood without heparin. Re-clotting was only observed in blood spiked with heparin at 5 U/mL without OsPA. The blood spiked with heparin at 10 or 20 U/mL did not clot without the addition of OsPA. Re-clotting occurred in blood spiked with heparin at 10 U/mL in samples containing 1 or 2 µg OsPA and with heparin at 20 U/mL in samples containing 1, 2 and 3 µg of OsPA. C) Comparison of blood clotting in the OsPA and the commercial BDRST tubes in recalcified citrated whole blood spiked with different concentrations of heparin by visual whole blood clotting assay. Note: no blood clotting was observed at 2000 s when the blood contained 5 U/mL heparin in BDRST tubes. D) Representative blood clotting traces and four parameters (R time (s), K time (s), Angle-α and MA value (mm)) from TEG assay that was carried out in parallel to the visual clotting assay for comparing the activity of OsPA and BDRST tubes in clotting recalcified citrated whole blood spiked with different concentrations of heparin shown in the above figure (Figure 3 C). N/A in the figures indicates that the parameters were not available in TEG assay. OsPA - prothrombin activator complex from the venom of *O. scutellatus*. BDRST – Becton Dickenson rapid serum tube. R – reaction time . K - clot formation time. MA – maximal amplitude. TEG – thromboelastography.

**Figure 5:**
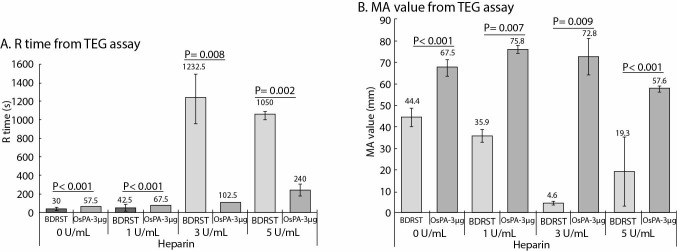
Comparison of two thromboelastography parameters (R time and MA value) in clotting of recalcified citrated whole blood spiked with different concentrations of heparin in tubes containing OsPA (3 µg/tube) and BDRST tubes. A) R time (s). B) MA value (mm). Data are the mean ± SD of four TEG assays (N = 4) for each concentration of heparin from two experiments, with each concentration done twice in one experiment. The Fisher exact test was used for testing the differences of both R times and MA value between OsPA and BDRST tubes. The P values are shown above the histograms of the two tubes. OsPA - prothrombin activator complex from the venom of O.scutellatus (Australian Coastal Taipan). BDRST – Becton Dickenson rapid serum tube. R- reaction time. MA – maximal amplitude. TEG – thromboelastography.

**Figure 6:**
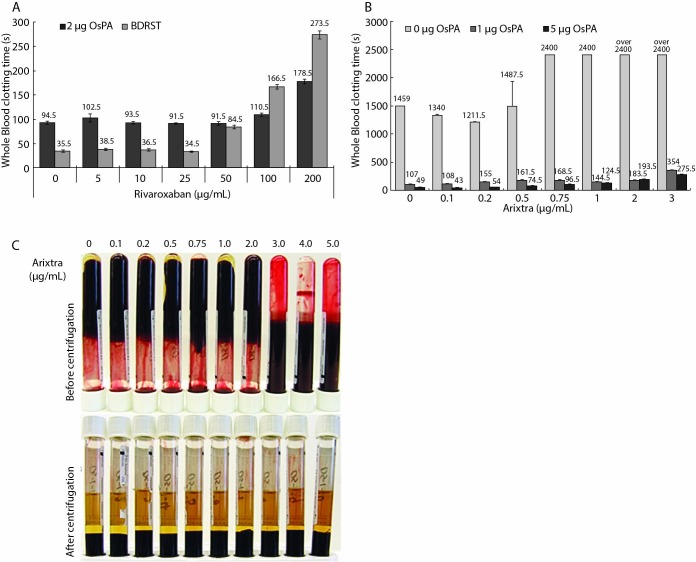
Activity of OsPA in clotting recalcified citrated whole blood spiked with different concentrations of two anticoagulants (Rivaroxaban and Arixtra) by visual whole blood clotting assay. A) Comparison of tube containing OsPA (2 µg/tube) and BDRST in clotting 4 mL of recalcified citrated whole blood spiked with increasing concentrationsof Rivaroxaban from 0-200 µg/mL. The clotting time for the recalcified citrated whole blood in the absence of either Rivaroxaban or OsPA was 3097.5 s (Supplementary figure 3). Data are the mean ± SD of duplicate assay. B) Activity of OsPA at 0, 3 and 5 µg/ tube in 4 mL of clotting recalcified citrated whole blood spiked with increasing concentrations of Arixtra from 0-3 µg/mL. In the absence of OsPA, the clotting times in recalcified citrated whole blood spiked with Arixtra at 2 or 3 µg/mL were over 2400 s. The data are the mean ± SD of duplicate assays. Figure 6 **(continued).** Activity of OsPA in clotting recalcified citrated whole blood spiked with different concentrations of two anticoagulants (Rivaroxaban and Arixtra) by visual whole blood clotting assay. C) Image shows 1 µg of OsPA in clotting 4 mL recalcified citrated whole blood spiked with different concentrations of Arixtra. 1 µg OsPA did not generate a rigid blood clot when the blood contained more than 3 µg/mL Arixtra. OsPA - prothrombin activator complex from the venom of *O.scutellatus*.

**Figure 7:**
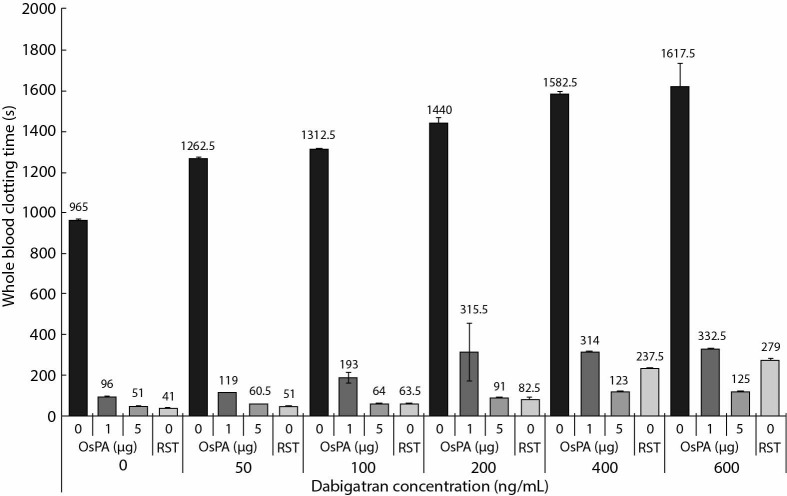
Activity of OsPA at 1 and 5 µg in clotting 4 mL of recalcified citrated whole blood spiked with different concentrations of dabigatran from 0-600 ng/mL. BDRST as for comparison was also used for clotting the recalcified citrated whole blood spiked with Dabigatran up to 600 ng/mL. In the figure, OsPA 1 and 5 µg/tube, RST = BDRST. The data are the mean ± SD of duplicate assays. OsPA - prothrombin activator complex from the venom of *O.scutellatus*. BDRST – Becton Dickenson rapid serum tube.

We showed that the clotting effectiveness of BDRST tubes was limited with increasing heparin concentration > 1 U/mL, likely due to insufficient thrombin concentration to overcome the heparin anticoagulant activity (Figure 2A), and hence latent clotting (occurring in the serum component after centrifugation) was observed in these tubes (Figure 2B) (*8*). In the absence of heparin, an OsPA amount as low as 0.0625 µg OsPA was capable of clotting 0.1 mL of plasma in 40.5 s (Figure 3). The 0.0625 µg of OsPA clotted 0.1 mL of plasma sample containing 1 and 2 U/mL of heparin at approximately 109.5 s and 135.9 s respectively and with 4U/mL of heparin it was 229.5 s (Figure 3), while in the absence of OsPA, heparin-spiked plasma failed to clot. Rapid clotting of re-calcified citrated whole blood by OsPA also occurred in heparin-spiked bloods across a heparin concentration range of 0-20 U/mL (Figure 4A). While lower amounts of OsPA (1-2 µg) clotted blood effectively containing 5 U of heparin/mL, 3 µg was required for efficient clotting of blood containing 10 and 20 U/mL of heparin/mL (Figure 4A). Re-clotting only occurred in the heparin-spiked blood at 5 U/mL without OsPA and at 10 or 20 U/mL with lower concentrations of OsPA (Figure 4B). When we compared these data to clotting in a commercial BDRST tube we failed to see any clotting at the higher concentrations of heparin in the thrombin tube. The results in Figure 4C, using visual clotting, reveal that no clot formation occurred in the BDRST tube containing 5 U/mL heparin and even at 3 U/mL clotting was weak (Supplementary figure 2). Use of the TEG assay for clotting supported in vitro clotting (Figure 4D). R times were significantly longer in the BDRST tube compared to the OsPA tube in the presence of increasing concentrations of heparin and MA values were significantly lower (Figure 5). We also demonstrated activity of OsPA in clotting recalcified citrated whole bloods spiked with other anticoagulants: Rivaroxaban, Arixtra, Clexane and Dabigatran at high concentrations. The results in Supplementary figure 3 show that OsPA succeeded in clotting blood spiked with Rivaroxaban at concentrations up to 20 µg/mL. Furthermore when comparison of clotting in the presence of Rivaroxaban was made between OsPA and BDRST tubes, clotting times were shorter in the BDRST tube over the range 0-25 µg/mL Rivaroxaban but at higher concentrations (50-200 µg/mL) the trend was reversed (Figure 6A), blood clots produced in both tubes were rigid. The results in Figure 6B show that OsPA at 5 µg/4 mL clotted effectively recalcified citrated whole blood in the presence of a second anticoagulant Arixtra, but OsPA at 1 µg/4 mL did not generate a rigid blood clot for the recalcified citrated whole blood containing Arixtra at 3 µg/mL or higher (Figure 6C), suggesting that higher amounts of OsPA are required for blood containing higher doses of Arixtra. The data for Clexane appears in Supplementary figure 4. For spiked samples, Dabigatran was incubated for 24h for conversion to the active metabolite. Results show that both OsPA at 1-5 µg/4 mL and BDRST tubes clotted effectively the recalcified citrated whole blood in the presence of Dabigatran, with rigid clots generated (Figure 7). Overall these data demonstrate the effectiveness of OsPA in clotting blood in the presence of a variety of anticoagulants and its superiority compared to clotting in a BDRST tube.

### Effectiveness of OsPA in clotting anticoagulated blood from patients

**Figure 8:**
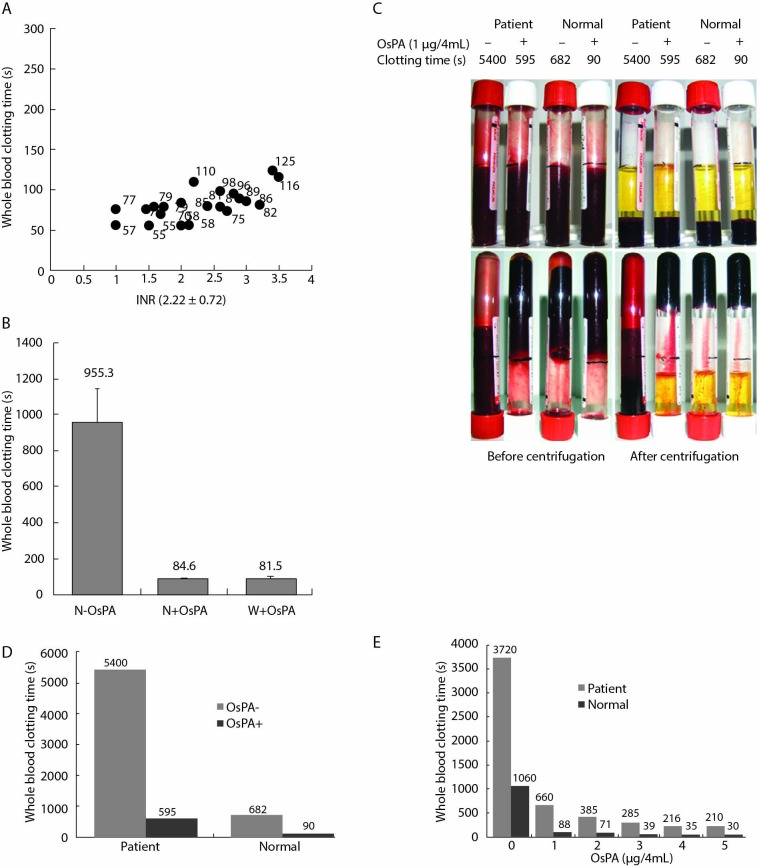
Activity of OsPA in clotting blood from patients on warfarin and those on Dabigatran treatment. A) Clotting activity of OsPA in recalcified citrated whole blood samples from 20 patients being treated with Warfarin at 1.0-3.55 of international normalised ratio (INR) with an average INR of 2.22 ± 0.72. With the addition of 1µg OsPA in 4 mL to warfarin bloods, the times for clotting the 20 patient blood samples ranged from 57 to 125 s. B) The mean clotting times (s) ± SD of normal recalcified citrated whole bloods without OsPA (N-OsPA, N = 35) and with 1 µg OsPA (N+OsPA, N = 35) in 4 mL blood samples; compared with that of the warfarin bloods (N = 20) with 1 µg OsPA (W+OsPA) in 4 mL blood samples. According to the one way ANOVA unequal variance analysis, the difference between N+OsPA and W+OsPA was not significant (P = 0.438). C) Clotting patterns of one Dabigatran-containing blood sample from a patient volunteer who was treated a no with the dabigatran with or without 1 µg OsPA in 4 mL blood samples, compared with that of normal blood. D) Clotting time of the dabigatran-containing blood sample from a patient volunteer who was treated with the dabigatran blood with or without 1 µg OsPA in 4 mL blood samples, compared with that of a normal blood. E) Clotting time of the dabigatran-containing blood sample from a patient volunteer who was treated with the dabigatran with different concentrations of OsPA in 4 mL blood, compared with that of a normal blood. OsPA - prothrombin activator complex from the venom of *O.scutellatus.*

Having shown that OsPA works effectively in clotting anticoagulated blood *in vitro*, we determined its effectiveness in clotting plasma and blood from patients on warfarin treatment. For warfarin whole blood, the clotting time for the 20 patients ranged from 57-125 s in the presence of 1 µg OsPA, while the clotting times in the absence of 1 µg OsPA were prolonged to over 4000 s (Figure 8A). The average clotting time for these patients were 81.5 ± 18.8, comparable to that for 35 healthy whole blood samples (84.6 ± 9.2) (Figure 8B). Based on unequal variance analysis, the clotting time of normal+OsPA blood was not significantly different from that of warfarin+OsPA blood (P = 0.438, Figure 8B). We also demonstrated that 1µg of OsPA clotted recalcified citrated plasma in less than 20 s for all patients. In another experiment, two healthy participants with INR 1.0-1.1 and two warfarin patients with INR of 2.5 and 4.7 were also recruited for plasma clotting assay by visual observation and TEG (Supplementary figure 5). The recalcified citrated plasma from the warfarin patients clotted at 46.5 s, compared to only 25.5 s from the healthy participants (Supplementary figure 5A). Furthermore, the TEG assay showed that the warfarin-containing blood (INR ≥ 4.7) with recalcification did not clot without the OsPA while that at INR 2.5 was extremely slow in clotting (Supplementary figure 5 B, C compared to two normal participants in Supplementary figure 5 D, E). These data clearly demonstrated that OsPA works efficiently in clotting blood from patients on warfarin.

We also determined the coagulant activities of OsPA in clotting blood from a patient treated with Dabigatran. Using the TEG assay, significantly longer R and K times were observed for the patient compared to control, demonstrating prolonged clotting times (Supplementary figure 6). A significantly reduced α-angle (27.9 compared to 62.9) indicated a high concentration of residual fibrinogen in the patient’s blood and a significantly lower MA value also indicated incomplete blood clotting (Supplementary figure 6). As expected, both PT and aPTT in plasma were significantly higher in the Dabigatran patient than the non-anticoagulated participant (Supplementary figure 1). We visually observed that the dabigatran-containing fresh blood sample from the patient in a BD-SST tube clotted in 5400 s but with a loose clot, whereas the control normal blood in the same tube was 682 s with complete clotting observed (Figure 8C). Addition of 1 µg OsPA to Dabigatran-containing fresh blood significantly reduced clotting time to 595 s, lower than the control normal blood, with a complete clot and no latent clotting in serum (Figure 8C). The clotting times of the fresh blood samples for both participants were significantly shortened with increasing concentration of OsPA (1-5 µg) *e.g.* at 3 µg OsPA the clotting time was 39 s for the control and 285 s for the patient (Figure 8D, E). These data demonstrate that rapid clotting can be produced from the dabigatran-containing blood with the addition of OsPA.

### Analyte stability in serum generated by OsPA and PtPA from anti-coagulated patients

We measured 31 analytes in serum samples from the seven participants, prepared in (Serum/OsPA) and (Serum/PtPA), compared with those samples prepared in (Serum/GBO) and the plasma samples prepared from (Plasma/Heparin tubes) (Table 1). In general, there was very good agreement between the analyte concentrations determined among the three forms of sera against the quality control (QC) (Supplementary table 3). Several analytes including total protein (TP), gamma-glutamyl transferase (GGT) and aspartate amino transferase (AST) showed variations up to 10% between these sera and the GRLH plasma but the differences were all within QC imprecision data (Supplementary table 3). The differences observed in AST are common findings between lithium heparin plasma and serum specimens and these analytes are expected to be higher in the serum samples due to the clotting process. The TP results were lower in the sera due to the prothrombin activators being able to remove the fibrinogen from the samples. The GGT differences were not analytically significant at this low level being within the analytical performance limits of the assay. However, non-parametric statistical analysis showed that there is no significant difference for 29 analytes among the four serum/plasma samples except for two analytes, triglyceride (Trig) and troponin I (Tnl). The triglyceride differences between the PtPA and OsPA tubes and the commercial serum tubes were due to storage of the prothrombin activator concentrates in 50% glycerol, since glycerol is a substrate in the triglyceride assay. Although the raw differences in TnI suggest that they were significant, the concentrations of five of the samples were very low (< 0.025 μg/L) and therefore less reliable.

## Discussion

We have demonstrated that OsPA is a very efficient procoagulant capable of rapidly promoting clotting of blood even in the presence of high levels of anticoagulants. OsPA was effective in the presence of a range of the most commonly used anticoagulants heparin, warfarin, Dabigatran, and Rivaroxaban that are widely used in up to 10% of patients in a tertiary hospital setting (*18*). This was the case both in *in vitro* experiments where blood was spiked with anticoagulant and when clotting blood from patients on anticoagulant therapy. As we described previously, OsPA-induced blood clotting is due to the initial and sustained generation of large amounts of thrombin that rapidly produces high quality serum for analyte detection, resulting in minimal residual fibrinogen in the serum (*5*). Snake venom prothrombin activators are also very effective in clotting mammalian blood as they are: a) constitutively active and thus fully active when isolated from venom; and b) partially or fully resistant to the coagulation cascade regulatory mechanisms, particularly to the actions of Protein C where for example the FV-like component of the Group C prothrombin activators is resistant to cleavage (*1*, *17*-*19*). This allows these prothrombin activators to continuously produce thrombin from endogenous prothrombin without control or de-activation, overwhelming antithrombin inhibition (and also any anticoagulants present). The technology also has the potential to reduce the number of blood collection tubes routinely used in the pre-analytical phase by isolating plasma initially for haematological analysis and subsequently serum after OsPA or PtPA treatment for immunological testing.

Commercially, the BDRST and Greiner BCA Fast clot tubes contain thrombin and are designed to rapidly clot blood samples. However, these tubes are not effective for patients on high heparin, with BDRST tubes not recommended for use in patients on heparin therapy (*6*, *19*). Heparin itself has anti-FXa activity as well as forming the heparin-antithrombin complex with its anticoagulant effect appearing to increase disproportionately in intensity and duration as the dose is increased (*20*). It is important to note that OsPA (3 µg) containing tubes not only clotted normal re-calcified citrated plasma and whole blood but also whole blood containing up to 20 IU of heparin/mL. Putting this into perspective, heparinised blood samples from anticoagulated patients in renal dialysis treatment usually contain concentrations in the range 0.3-1.0 U/mL, while in the case of patients undergoing cardiopulmonary bypass (CPB) the heparin concentration is up to 10.0 U/mL (*12*, *21*). OsPA (1 µg) containing tubes were also effective in clotting blood from patients on warfarin therapy across a wide range of INRs. Visual clotting and TEG experiments using BDRST tubes also demonstrated efficient clotting of plasma samples from warfarin patients albeit with weaker MA values (Supplementary figure 5 B,C). This is consistent with previous results that Australian snake venoms clotted warfarinised blood as they are able to activate the descarboxy form of prothrombin formed (*22*). Dabigatran is an oral anticoagulant offering an alternative to warfarin for use in preventing stroke in patients with atrial fibrillation (*23*). This compound inhibits thrombin by binding to its active site with high affinity (*24*). In this study, we demonstrated that a small amount of OsPA (1 µg) can significantly reduce the clotting times of Dabigatran-containing blood leading to a complete clot and the generation of good quality serum.

In addition, comparison between OsPA-containing tubes and commercial tubes showed that the presence of OsPA did not interfere with serum analyte determinations. Quality control has shown that the analyte concentrations determined in sera from OsPA- and PtPA-containing tubes were consistent with those in serum from the GBO-RST tube. These data demonstrate that OsPA works efficiently in clotting blood from anticoagulated patients and is suitable for the generation of high quality serum for analyte determination in a clinical setting. Worldwide sales of anticoagulants have been predicted to have doubled between 2011 and 2018 (*25*). This in turn will challenge current commercial blood collection tubes to clot these samples and produce high quality serum in a timely manner. OsPA snake venom prothrombin activator technology which efficiently clots blood from patients on anticoagulant therapy has the capacity to meet these demands to produce quality serum.

Plasma samples produced from heparinised blood collection tubes have been used as an alternative in the analysis of many common chemical analytes (*26*). Plasma tubes are thought to have some advantages over blood serum tubes as they can be centrifuged immediately to produce plasma with higher yield (*27*). When repeated studies were carried out, measuring several key analytes of sera stored at room temperature or 4°C for 0, 8, 24 and 36 hours, it is expected that there would be significant differences in stability and reproducibility of these four clinically relevant analytes between plasma/heparin and serum/PtPA samples, even between serum/GBO-SST and serum/PtPA samples. The expectation would be that high quality sera would show minimal variability as compared to plasma analyte data (*28*). Plasma/heparin samples were clearly unstable because blood cells present in the plasma lyse over time or are damaged when transferred for analysis, affecting the analytes in the sample (*29*). Furthermore, some analyte assays require sera especially when testing for autoantibodies such as T3/T4 (*30*). In contrast, serum/PtPA samples were typically the most stable on storage. As a result, there were significant changes of the four analytes that exceed the LSC limits in the plasma/heparin samples, while it only had small changes within the LSC limits in serum/PtPA samples. While we observed triglyceride differences in the PtPA and OsPA tubes compared to the commercial serum tubes, this was due to storage of the prothrombin activator concentrates in glycerol because glycerol interferes in the triglyceride assay we employed, use of other assays for triglycerides would have addressed this problem (*31*). In clinical settings a diagnosis can be made by combining other diagnostic results together with analytical results and the history of the patient (*32*). Thus, lack of agreement of quantitative concentration and accuracy of even one analyte among the specific time points in the plasma/heparin samples indicates the unreliability of plasma/heparin. Thus, serum prepared from patients on anticoagulant therapy in the presence of prothrombin activators (OsPA or PtPA) is suitable for accurate analyte determination.

In conclusion, we have demonstrated that snake venom prothrombin activators (OsPA and PtPA) overcome the blood clotting problems associated with use of anticoagulants. OsPA containing blood collection tubes can clot effectively anticoagulated blood and generate high quality serum from this blood within 5 min. Analyte determinations have revealed that there is an excellent agreement of analytical data on serum samples prepared using OsPA and PtPA and those using commercial serum and plasma tubes. Thus, this technology has great potential to reduce the numbers of blood collection tube routinely with faster turn-around-times.

## Supplementary material

Supplementary material
