# Supplementary material for: Rapid serum tube technology overcomes problems associated with use of anticoagulants
Source: Biochem Med (Zagreb). 2019 Oct 15;29(3):030706. doi: 10.11613/BM.2019.030706 (PMC6784418; doi:10.11613/BM.2019.030706)
Supplement: Supplementary file 1 — Supplementary material [file bm-29-3-030706-S1.pdf]

Supplementary table 1. Information of dabigatran patient for blood collection

**Dabigatran patient**	**SE**
Dabigatran dose	Twice a day: Morning and evening<br>110 mg/*per* tablet
Date: 25th Sept 2014	Taking dabigatran at 10:30 am<br>Blood collection at 2:30 pm
Date: 1st October 2014	Taking dabigatran at 9:30 am<br>Blood collection at 2:30 pm
**Normal blood control**	**PM**

Supplementary table 2. Quality Control (QC) imprecision on the Beckman DxC800 and DxI800 analysers (*DxI800) analysers, Chemical Pathology, Princess Alexandra Hospital

Analyte	QC Level 1			QC Level 2			QC Level 3		
	Mean	SD	CV %	Mean	SD	CV %	Mean	SD	CV %
**Na**	132	1.27	1.0	150	1.33	0.9			
**K**	3.9	0.06	1.4	6.0	0.07	1.2			
**Cl**	85	1.17	1.4	100	1.19	1.2			
**HCO_3**	17	0.66	3.8	29	0.85	2.9			
**Glu**	4.8	0.12	2.6	16.7	0.28	1.8			
**Urea**	5.2	0.17	3.3	15.9	0.41	2.6			
**Crea**	68	3.34	4.9	491	8.93	1.8			
**Urate**	0.23	0.001	1.9	0.49	0.01	1.5			
**TP**	41	0.73	1.8	67	1.07	1.6			
**Alb**	26	0.37	1.4	40	0.54	1.3			
**TBIL**	21	1.37	6.4	93	1.96	2.1			
**ALP**	106	2.79	2.6	471	7.93	1.7			
**GGT**	38.7	2.03	5.3	155	2.88	1.9			
**ALT**	25	1.52	6.2	94	2.00	2.1			
**AST**	34	1.35	3.9	201	2.39	1.2			
**LD**	149	3.21	2.2	408	5.65	1.4			
**CK**	139	2.53	1.8	459	5.8	1.3			
**Ca**	2.07	0.03	1.6	2.86	0.04	1.3			
**Phos**	1.00	0.02	1.9	2.86	0.04	1.5			
**Mg**	0.84	0.02	2.5	1.63	0.04	2.3			
**Lipase**	29	2.39	8.2	57	4.05	7.1			
**Chol**	3.0	0.05	1.7	6.5	0.15	2.3			
**Trig**	1.0	0.04	3.6	2.0	0.05	2.7			
**HDL**	1.1	0.04	3.5	1.9	0.05	2.6			
**Fe**	10	0.29	2.8	34	1.93	5.7			
**Trsf**	1.0	0.04	3.5	2.3	0.08	3.5			
**CRP**	4.6	0.36	7.9	10.0	0.55	5.4			
**TnI**	0.052	0.01	16.1	0.55	0.04	7.9	10.2	0.69	6.7
**Cortisol**	139	8.85	6.4	552	28.48	5.2	906	41.7	4.6
**fT4**	7.51	0.62	8.2	23.93	0.94	4.0	50.9	1.92	3.8
**fT3**	3.49	0.23	6.5	7.38	0.36	4.9	12.76	0.68	5.4
**TSH**	0.71	0.05	7.1	4.17	0.26	6.3	23.1	1.53	6.6
**Ferritin**	19	1.68	8.8	159	9.67	6.1	358	22.42	6.3

Note: Laboratory quality control is designed to detect, reduce, and correct deficiencies in a laboratory's internal analytical process prior to the release of patient results. Samples from the 50 participants were loaded on the same Beckman instruments at the same time and within 2 hours post-centrifugation, except where recurrent latent clotting was encountered. The analytes tested plus semi-quantitative haemolysis levels are listed in the result tables. The upper limits of imprecision of the between-run coefficients of variation (CVs) from the two and three internal quality control concentrations for the analytes tested on the Beckman DxC800 analysers.

To calculate the acceptable change limit for each assay or the least significant change (LSC) which is considered to be smallest difference between successive measurements that are real the following equation was used: LSC = 2.77 $\sqrt{}CV_{biological\ difference} + CV_{analytical\ difference})$ to determine if analytical differences existed between the paired differences. With the samples being collected at the same time in the different tubes, no biological variation needed to be included in the calculation of the least significant change, therefore LSC = 2.77 $\sqrt{}CV_{analytical\ difference}$. The analytical coefficient of variation (ACV) for each assay was obtained from Table 2. The ACV used in calculating the LSC was the one that most closely matches the mean of the participants result set. The critical allowable limits (CAL) were adopted from a publication by Tanner *et al* .

Na – sodium. K – potassium. Cl – chloride. HCO3 - carbon dioxide.Glu – glucose. Crea – creatinine.TP - total protein. Alb – albumin. TBIL - total bilirubin. ALP - alkaline phosphatase. GGT - gamma glutamyltransferase. ALT - alanine aminotransferase. AST -aspartate aminotranferase. LD - lactate dehydrogenase. CK - creatinine kinase. Ca – calcium. Phos - inorganic phosphate. Mg – magnesium. Chol – cholesterol. Trig – triglyceride. DHL - high density cholesterol. Fe – iron. Trsf – transferrin. CRP - C-reactive protein. TnI- troponin I. fT4 - free thyroxine. fT3 - free triiodothyronine. TSH - thyroid stimulating hormone.

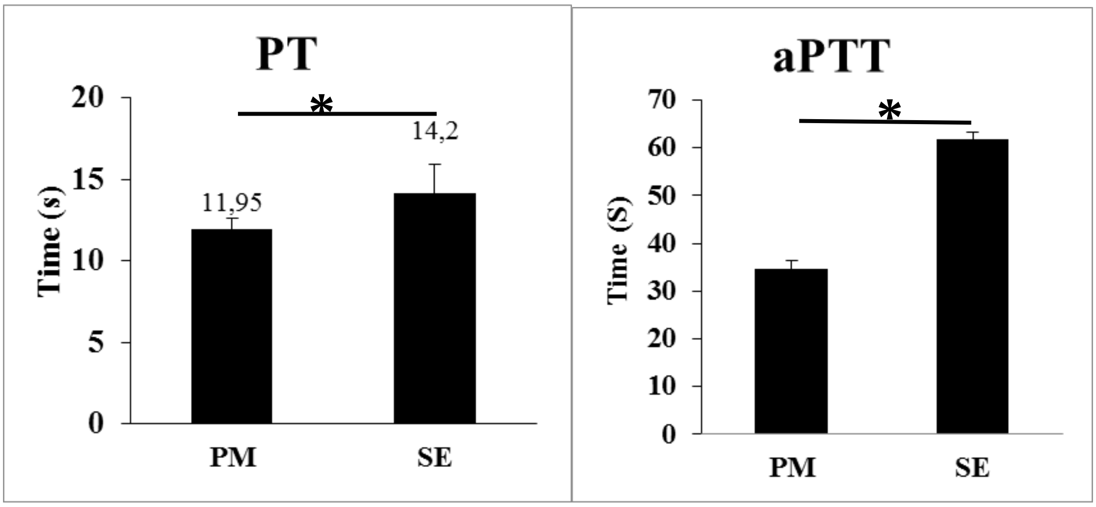

Supplementary figure 1. Prothrombin time (PT) and activated partial thromboplastin time (aPTT) of dabigatran patient plasma compared with those of normal plasma. The data are the mean ± standard deviation (SD) of triplicate assays. *represents that the difference between two participants was significantly different at $P < 0.05$, respectively.

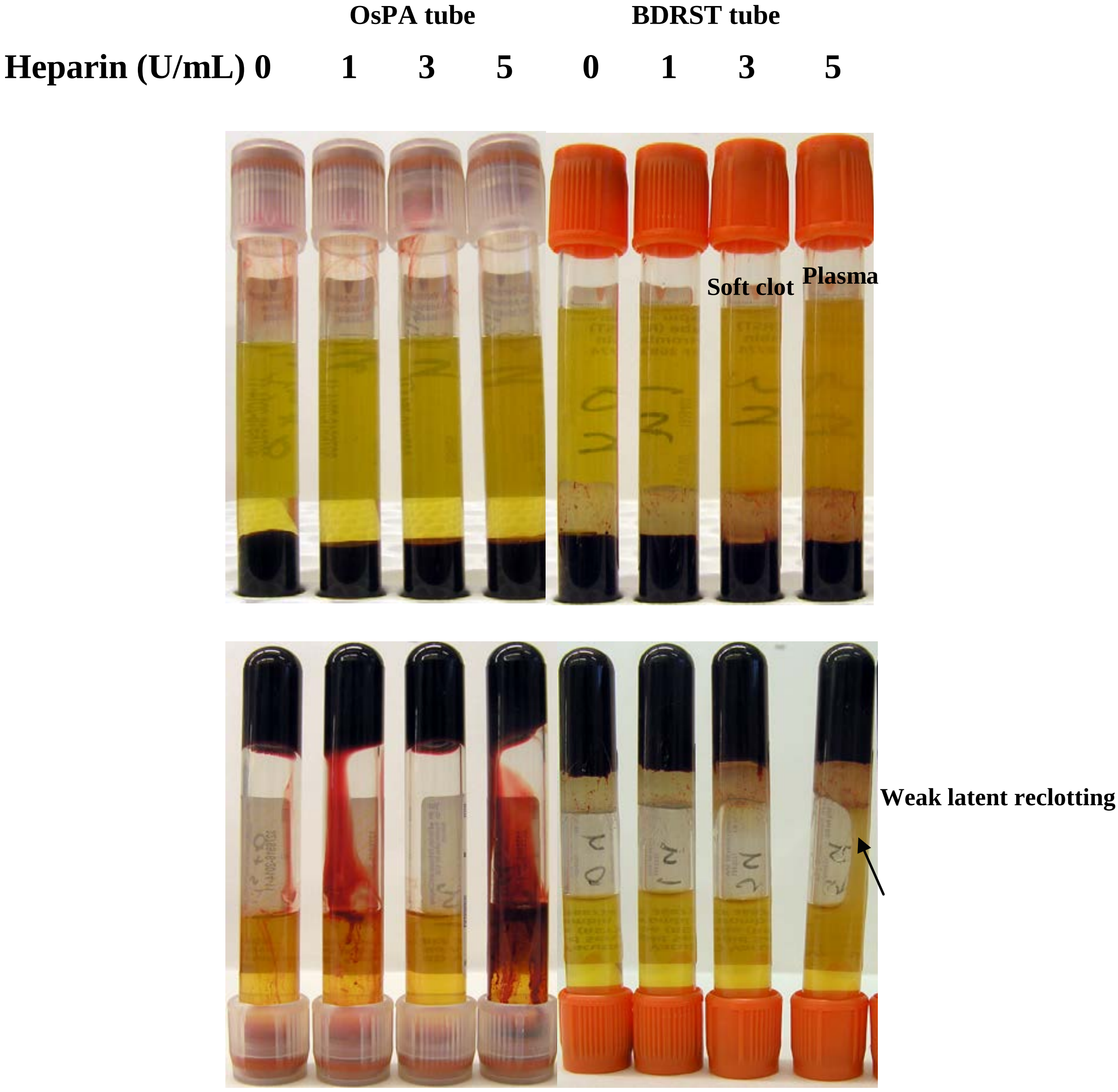

Supplementary figure 2. Images of serum/plasma in OsPA (3 µg/tube) tube and BDRST tube in clotting recalcified citrated whole blood spiked with different doses of heparin by TEG assay. Upper panel: Images of blood containing different doses of heparin in OsPA tube and BDRST tube after the blood in tubes for 2000 sec whether they were clotted or not were centrifuged at 3000 rpm for 10 min. Lower panel: Upside-down images of blood containing different doses of heparin in OsPA containing tubes and BD RST tubes at 18 hours on bench after centrifugation. Only plasma was produced in BDRST tube in clotting blood containing 5 U/mL heparin

and weak latent reclotting was observed at 18 h post-centrifugation. OsPA - prothrombin activator complex from the venom of *O. scutellatus*. TEG – thromboelastography. BDRST- Beckton Dickenson rapid serum tube.

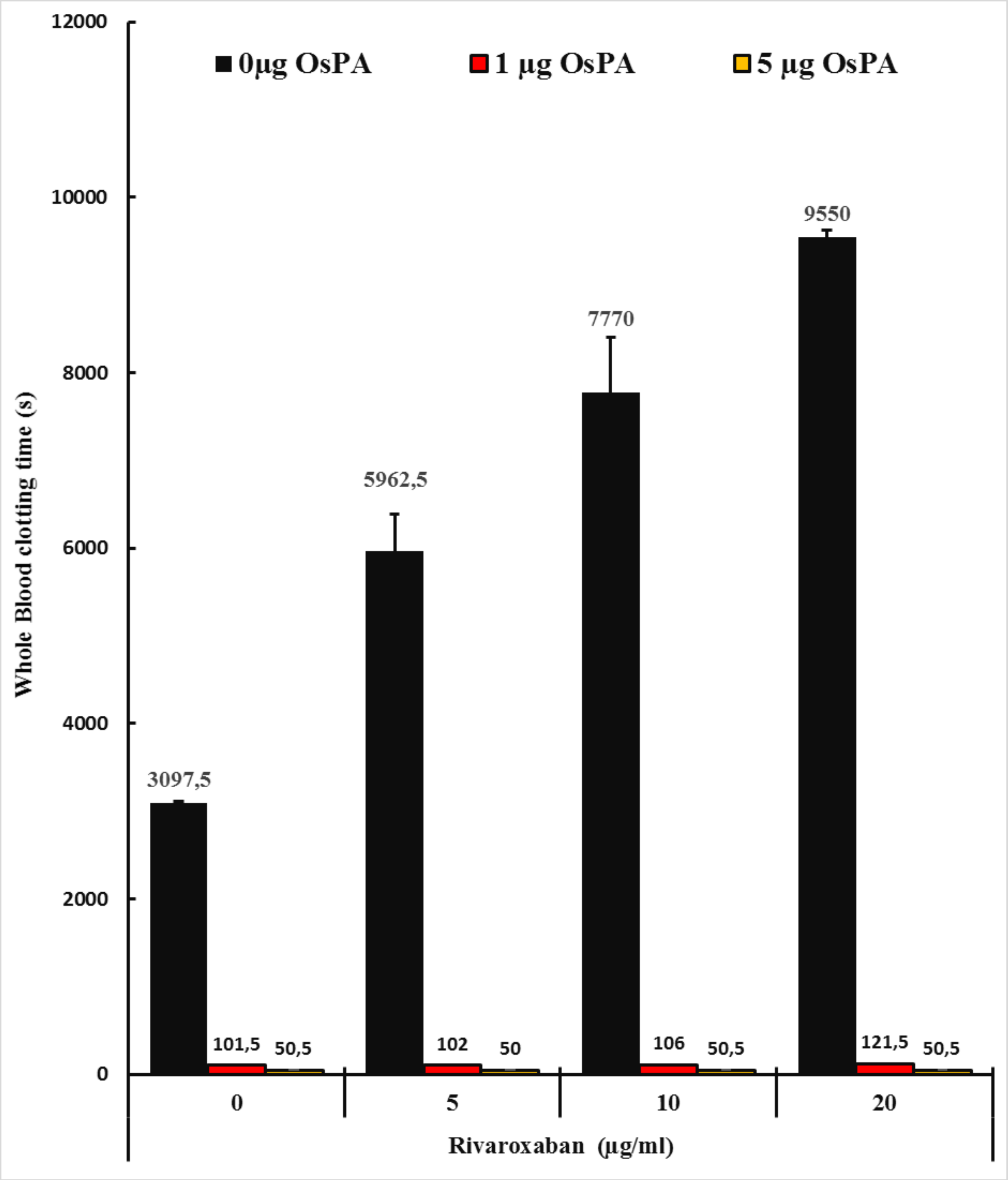

Supplementary figure 3. Activity of OsPA in clotting recalcified citrated whole blood spiked with different concentrations of Rivaroxaban. In the absence of OsPA, the recalcified citrated whole blood spiked with Rivaroxaban over 80 µg/mL was clotted over 2 hours (14400 s). The data are the mean ± standard deviation (SD) of

duplicate assays. OsPA - prothrombin activator complex from the venom of *O. scutellatus.*

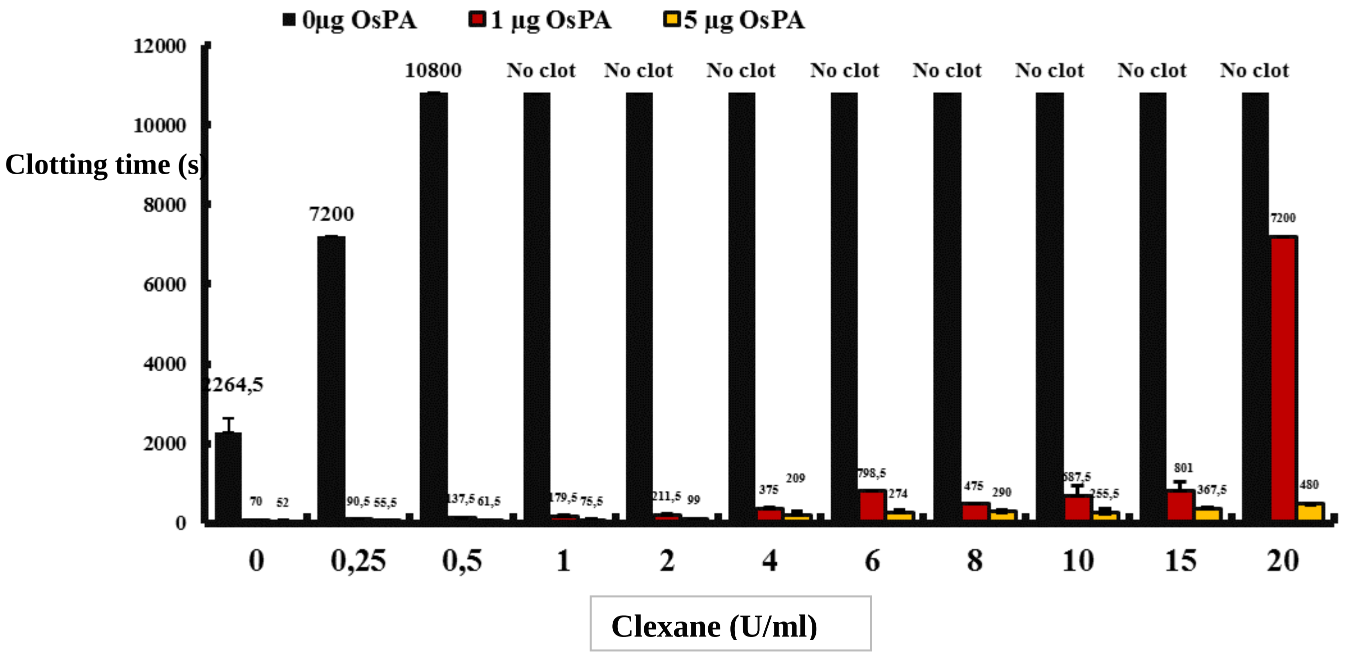

Supplementary figure 4. Activity of OsPA in clotting recalcified citrated whole blood spiked with different concentrations of Clexane. In the absence of OsPA, the recalcified citrated whole blood spiked with Clexane over 1 U/mL did not clot. The data are the mean ± standard deviation (SD) of duplicate assays. OsPA - prothrombin activator complex from the venom of *O. scutellatus*.

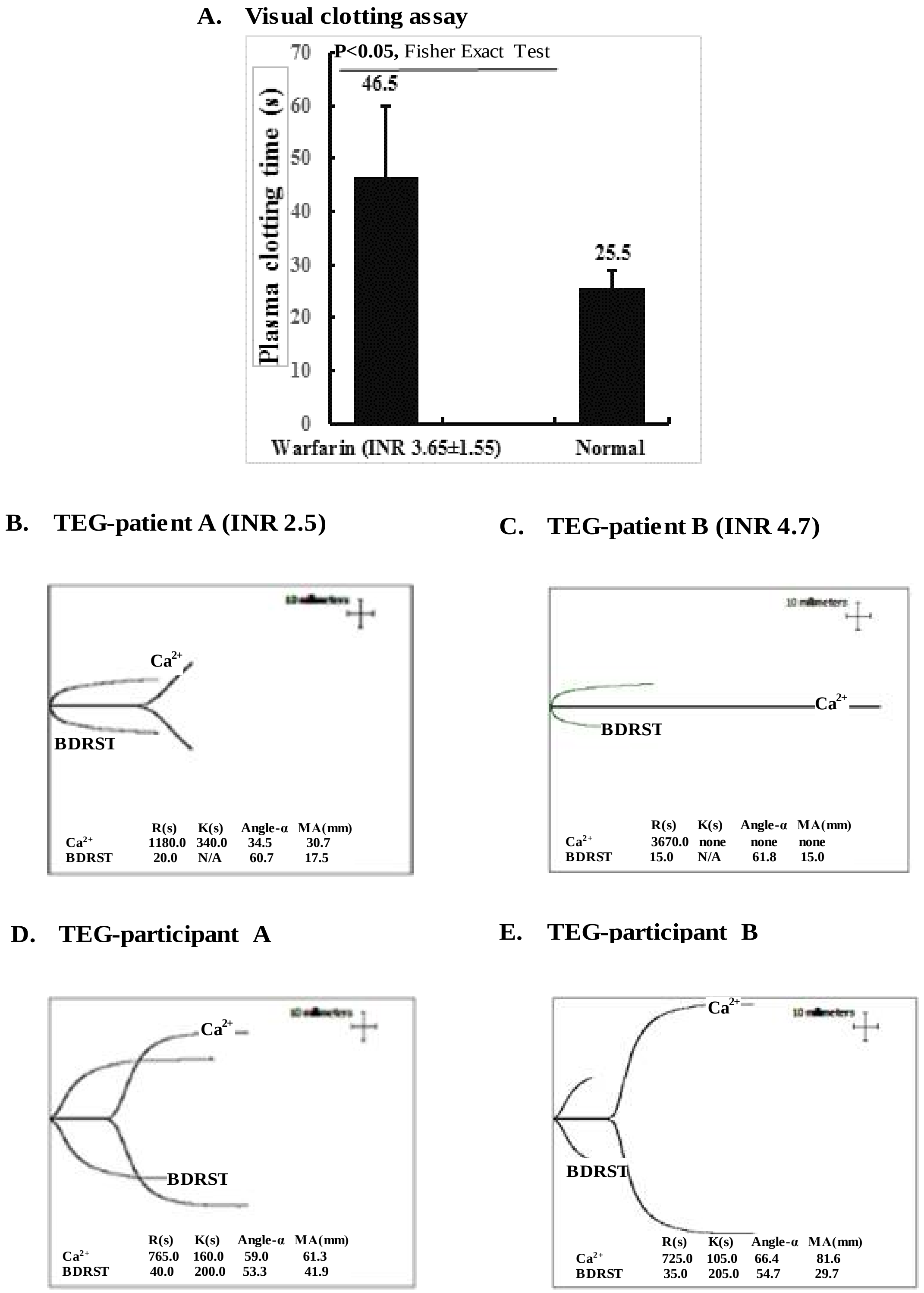

Supplementary figure 5. Activity of commercial thrombin activator tubes (BDRST) in clotting recalcified citrated plasma from two warfarin patients and two normal participants by visual clotting and TEG assays. **A.** Visual clotting results,

the data are the mean ± standard deviation (SD) (N = 2); Fisher Exact test showing $P < 0.05$ between warfarin patients and normal participants. **B and C.** TEG for two warfarin patients. **D and E.** TEG for two normal participants. TEG – thromboelastography.

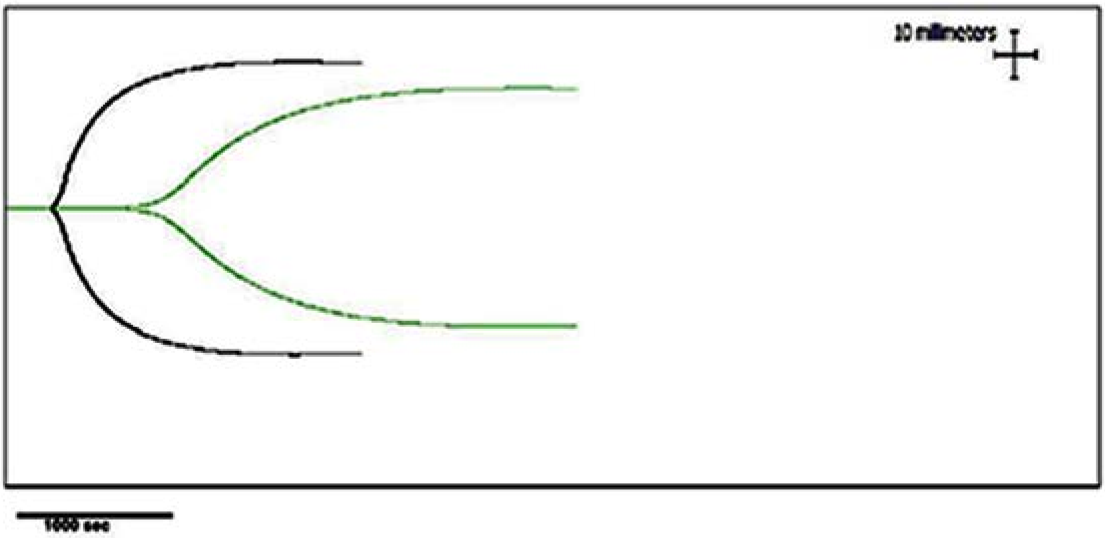

Tracings from top to bottom:

Seq	Channel	Patient	ST	Sample description			Operator	Machine SN
1	2	PM blood recalcified 8/10/2014	--	320 uL blood, 20 uL Ca	8/10/2014	12:51 PM	Temporary	03d71572
2	1	SE blood recalcified	--	320 uL blood, 20 uL Ca	1/10/2014	03:37 PM	Temporary	03d71572

Seq	Channel	R sec	K sec	Angle deg	MA mm	PMA	G Kd/sc	EPL %	A mm	CI	LY30 %
1	2	370.0	115.0	62.9	62.8		8.4	"0.0"	62.6		"0.0"
2	1	1,070.0	430.0	27.9	50.4		5.1	"0.0"	51.5		"0.0"

Supplementary figure 6. TEG assay shows clotting characteristics of citrated whole blood samples from dabigatran patient and normal participant. Green trace is representative of dabigatran patient and black trace is representative of health participant. The dabigatran patient has significantly higher R and K times and lower angle and MA values. R – reaction time. K - clot formation time. MA – maximal amplitude. TEG – thromboelastography.
